# Uremia Preventing Osmotic Demyelination Syndrome Despite Rapid Hyponatremia Correction

**DOI:** 10.1177/2324709620918095

**Published:** 2020-05-15

**Authors:** Srinadh Annangi, Snigdha Nutalapati, Srikanth Naramala, Pradeep Yarra, Khalid Bashir

**Affiliations:** 1 Morehouse School of Medicine, Atlanta, GA, USA; 2 Adventist Medical Center, Hanford, CA, USA; 3 University of Kentucky, Lexington, KY, USA

**Keywords:** hyponatremia, dialysis, uremia, myelinolysis, cerebral edema

## Abstract

Hyponatremia is the most common electrolyte abnormality encountered both in the inpatient and outpatient clinical settings in the United States. Rapid correction leads to a deranged cerebral osmotic gradient causing osmotic demyelination syndrome. Coexisting azotemia is considered to be protective against osmotic demyelination syndrome owing to its counteractive effect on osmolarity change that occurs with rapid hyponatremia correction. In this article, we report the case of a 37-year-old male who presented with altered mentation, acute azotemia, and severe electrolyte derangements, with serum blood urea nitrogen 160 mg/dL, creatinine 8.4 mg/dL, sodium 107 mEq/L, potassium 6.1 mEq/L, bicarbonate 7 mEq/L, and anion gap of 33. Given refractory hyperkalemia with electrocardiogram changes, emergent dialysis was performed. Despite our efforts to avoid rapid correction, serum sodium was corrected to 124 mEq/L and blood urea nitrogen decreased to 87 mg/dL at the end of the 5-hour dialysis session. Fortunately, hospital course and 4-week post-discharge clinic follow-ups were uncomplicated with no neurological sequela confirmed by neurological examination and magnetic resonance imaging.

## Introduction

Osmotic demyelination syndrome (ODS), formerly called as central pontine myelinolysis from rapid hyponatremia correction, was first described by Adams et al.^1^ Hyponatremia is the most common electrolyte abnormality encountered in the clinical practice with a reported prevalence of 9% to 31% for a serum sodium (Na) <130 mEq/L and 0.03% to 0.49% for serum Na <116 mEq/L.^2,3^ A study with National Health and Nutrition Examination Survey data reported a prevalence of 1.72% among the United States general population.^4^ The annual health care burden of hyponatremia is estimated to be 1.6 billion to 3.6 billion dollars.^5^ Human brains adaptation to reduce the risk of cerebral edema from hypotonic hyponatremia itself makes it vulnerable to osmotic stress that develops from rapid hyponatremia correction. The incidence of ODS is reported to be very low at 0.5% to 2% in the general population and 10% among patients undergoing liver transplantation.^6-9^ Most occurred among subjects with a serum sodium <120 mEq/L and a sodium correction of >10 to 12 mEq/L in the initial 24 hours of presentation.^8-12^ Despite its low incidence, given significant morbidity and mortality associated, it is still recommended to avoid the rapid correction of hyponatremia. The recommended rate of hyponatremia correction is 4 to 6 mEq/L in 24 hours. Among subjects with severe symptomatic hyponatremia, this correction should be achieved in the initial 4 to 6 hours with a maximum correction rate not exceeding 8 mEq/L within 24 hours.^13,14^ We hereby report a case of azotemia with severe hyponatremia in which sodium correction occurred rapidly devoid of any neurological sequelae.

## Case Report

A 37-year-old man with a past medical history of pancreatitis, type 2 diabetes mellitus on insulin, and ulcerative colitis was brought to the emergency department with complaints of nausea, vomiting, and diarrhea for a 1-week duration followed by anuria for 48 hours prior to this presentation. Vital signs on presentation include a temperature of 36.3°C, blood pressure of 103/60 mm Hg, heart rate of 79 beats per minute, and respiratory rate of 16 breaths per minute, saturating at 100% on room air. On examination, the patient appeared somnolent with dry mucous membranes and loss of skin turgor. Limited neurologic examination was unremarkable. Comprehensive serum drug screen was negative. Laboratory data are relevant for serum blood urea nitrogen 160 mg/dL, creatinine 8.4 mg/dL, Na 107 mEq/L, potassium 6.1 mEq/L, bicarbonate 7 mEq/L, anion gap of 33, glucose 169 mg/dL, and measured serum osmolality of 297 mOsm/L. Electrocardiogram showed normal sinus rhythm with peak T-waves. Urine studies revealed Na <10 mEq/L, creatinine 286 mg/dL, and osmolality of 311 mOsm/L, with hyaline casts and fractional excretion of sodium of 0.6%. No prior history of renal disease noted, and renal ultrasound was unremarkable. Given refractory and worsening hyperkalemia, worsening uremia, and acidosis, emergent dialysis was planned. However, the lowest sodium bath available for dialysis being 130 mEq, 0.45% normal saline was infused during the dialysis session to prevent rapid sodium correction. He received one session of hemodialysis and serum Na increased to 124 mEq/L and blood urea nitrogen decreased to 87 mg/dL during the 5-hour dialysis session. The patient’s mentation gradually improved, fully alert, awake, and oriented without any immediate neurological deficits. Detailed neurological examination performed prior to discharge was also unremarkable, including normal cranial nerve examination, muscle strength, and deep tendon reflexes, sensory function, gait, and coordination. Both T1- and T2-weighted imaging sequences on magnetic resonance imaging (MRI) scan performed 7 days after the rapid correction event was unremarkable. The patient’s neurological examination remained unremarkable on his 4-week posthospital clinic follow-up.

## Discussion

Osmotic demyelination syndrome or osmotic myelinolysis was first described in alcoholics and in malnourished by Adams et al in 1959; however, the etiological role of sodium was not identified at that time.^1^ The etiological role of sodium correction rate in causing myelinolysis was confirmed later in animal models.^15^ Serum Na is the primary determinant of serum tonicity. As hyponatremia develops, brain cells adapt by the efflux of organic and inorganic osmolytes to avoid relative hypotonicity. Efflux of inorganic osmolytes like potassium and chloride occurs within minutes to hours, which is followed by a delayed efflux of organic osmolytes like amino acids over the next few days.^16,17^ During the hyponatremia correction, brain cells should adapt to relative hypertonicity caused by increasing extracellular sodium concentrations. This adaption occurs by the influx of osmolytes back into brain cells. As organic solutes take longer time to reaccumulate, in the event of a rapid correction, delayed reaccumulation of organic solutes leads to a relatively hypertonic serum compared with brain cells eventually causing an efflux of water leading to ODS.^18^

On the other hand, rapid correction of azotemia in dialysis leads to cerebral edema owing to slower diffusion of urea across the blood-brain barrier.^19^ In animal studies, the presence of concurrent azotemia decreased the incidence and severity of myelinolysis with rapid hyponatremia correction.^16^ Acute azotemia being hypertonic state triggers the intracellular accumulation of organic osmolytes as an adaptive mechanism to prevent water influx into brain cells.^20^ This also leads to the synthesis of transporters for organic osmolytes facilitating rapid redistribution with azotemia correction.^21^ The increased intracellular concentration of cerebrospinal fluid urea and other organic osmolytes seen with azotemia state partially counteracts the extracellular hyperosmolarity that develops with rapid hyponatremia correction.

The above-mentioned counteracting mechanism of azotemia and hyponatremia on cerebral osmolarity, when coexist, was postulated to have a possible protective effect in preventing ODS from rapid sodium correction.^16,21,22^ One such observation was from retrospective analysis done by Dhrolia et al supporting the protective effect of azotemia.^23^ In their retrospective analysis, none of their 52 subjects with azotemia and hyponatremia developed ODS despite a mean 24-hour sodium correction by 15.4 (±3.4). However, in this analysis, neurological examination was reported immediately after dialysis and only 2 out of 52 subjects had MRI performed. MRI is the recommended imaging to diagnose ODS. Characteristic findings include symmetric signal hyperintensity in the central pons on T2-weighted and fluid attenuation inversion recovery sequences with corresponding decreased T1-weighted signal.^24,25^ Clinical manifestations of myelinolysis are usually not apparent immediately and are often delayed by up to 5 to 6 days after rapid sodium correction. Absence of MRI findings and delayed neurologic examination in that study, it would be a dangerous assumption to conclude that uremia had prevented ODS among all their study subjects.^26-28^ Fortunately, our case did not have any neurologic sequelae confirmed by delayed neurological testing and MRI imaging. Nevertheless, not all patients with azotemia and hyponatremia can be protective against ODS and clinical models to pinpoint at-risk populations for ODS are not yet developed. ODS among uremic subjects with rapid sodium correction has been reported and confirmed by delayed neurological examination and MRI.^26,29-31^ Until further evidence, it will be premature to conclude that uremia prevents ODS, as this might lead a false sense of assurance among the physicians and not to follow any preventive measures to avoid rapid sodium correction. Given the detrimental effects of ODS and lack of sustained evidence on the protective effect of azotemia, physicians should follow all possible precautions to avoid rapid sodium correction. Few such measures include reduction of dialysate sodium, infusion of hypotonic saline with dialysis, increasing dialysis duration, or perform intermittent short duration sessions if clinically feasible.

Osmotic demyelination syndrome is a rare preventable complication of rapid hyponatremia correction and is associated with significant morbidity and mortality. Though azotemia was proposed to be protective, not all previously reported cases with azotemia were fortunate enough to be exempt from developing ODS. Given the detrimental effects of this preventable condition, until further evidence, physicians should still follow all the necessary steps to avoid rapid hyponatremia correction even among cases with concurrent azotemia.

## References

[1] Adams RD Victor M Mancall EL. Central pontine myelinolysis: a hitherto undescribed disease occurring in alcoholic and malnourished patients. AMA Arch Neurol Psychiatry. 1959;81:154-172. 13616772

[2] Hawkins RC. Age and gender as risk factors for hyponatremia and hypernatremia. Clin Chim Acta. 2003;337:169-172. 14568195 10.1016/j.cccn.2003.08.001

[3] Upadhyay A Jaber BL Madias NE. Epidemiology of hyponatremia. Semin Nephrol. 2009;29:227-238. doi:10.1016/j.semnephrol.2009.03.004 19523571

[4] Mohan S Gu S Parikh A Radhakrishnan J. Prevalence of hyponatremia and association with mortality: results from NHANES. Am J Med. 2013;126:1127-37. doi:10.1016/j.amjmed.2013.07.021 PMC3933395 24262726

[5] Boscoe A Paramore C Verbalis JG. Cost of illness of hyponatremia in the United States. Cost Eff Resour Alloc. 2006;4:10. doi:10.1186/1478-7547-4-10 16737547 PMC1525202

[6] Kleinschmidt-Demasters B Rojiani AM Filley CM. Central and extrapontine myelinolysis: then . . . and now. J Neuropathol Exp Neurol. 2006;65:1-11. 16410743 10.1097/01.jnen.0000196131.72302.68

[7] Lampl C Yazdi K. Central pontine myelinolysis. Eur Neurol. 2002;47:3-10. 11803185 10.1159/000047939

[8] George JC Zafar W Bucaloiu ID Chang AR. Risk factors and outcomes of rapid correction of severe hyponatremia. Clin J Am Soc Nephrol. 2018;13:984-992. 29871886 10.2215/CJN.13061117 PMC6032596

[9] Ayus JC Krothapalli RK Arieff AI. Treatment of symptomatic hyponatremia and its relation to brain damage. N Engl J Med. 1987;317:1190-1195. 3309659 10.1056/NEJM198711053171905

[10] Geoghegan P Harrison AM Thongprayoon C. Sodium correction practice and clinical outcomes in profound hyponatremia. Mayo Clin Proc. 2015;90:1348-1355. doi:10.1016/j.mayocp.2015.07.014 26434962

[11] Aegisdottir H Cooray C Wirdefeldt K Piehl F Sveinsson O. Incidence of osmotic demyelination syndrome in Sweden: a nationwide study. Acta Neurol Scand. 2019;140:342-349. 31343728 10.1111/ane.13150

[12] Sterns RH. Severe symptomatic hyponatremia: treatment and outcome: a study of 64 cases. Ann Intern Med. 1987;107:656-664. 3662278 10.7326/0003-4819-107-5-656

[13] Verbalis JG Goldsmith SR Greenberg A Schrier RW Sterns RH. Hyponatremia treatment guidelines 2007: expert panel recommendations. Am J Med. 2007;120(11 suppl 1):S1-S21. 17981159 10.1016/j.amjmed.2007.09.001

[14] Sterns RH Nigwekar SU Hix JK. The treatment of hyponatremia. Semin Nephrol. 2009;29:282-299. doi:10.1016/j.semnephrol.2009.03.002 19523575

[15] Kleinschmidt-DeMasters BK Norenberg MD. Rapid correction of hyponatremia causes demyelination: relation to central pontine myelinolysis. Science. 1981;211:1068-1070. 7466381 10.1126/science.7466381

[16] Soupart A Penninckx R Stenuit A Decaux G. Azotemia (48 h) decreases the risk of brain damage in rats after correction of chronic hyponatremia. Brain Res. 2000;852:167-172. 10661508 10.1016/s0006-8993(99)02259-3

[17] Sterns RH Baer J Ebersol S Thomas D Lohr JW Kamm DE. Organic osmolytes in acute hyponatremia. Am J Physiol. 1993;264(5 pt 2):F833-F836. 8498536 10.1152/ajprenal.1993.264.5.F833

[18] Verbalis JG Gullans SR. Rapid correction of hyponatremia produces differential effects on brain osmolyte and electrolyte reaccumulation in rats. Brain Res. 1993;606:19-27. 8096428 10.1016/0006-8993(93)91564-9

[19] Kennedy AC Linton AL Eaton JC. Urea levels in cerebrospinal fluid after haemodialysis. Lancet. 1962;1:410-411. 14455152 10.1016/s0140-6736(62)91365-x

[20] Trachtman H Futterweit S Tonidandel W Gullans SR. The role of organic osmolytes in the cerebral cell volume regulatory response to acute and chronic renal failure. J Am Soc Nephrol. 1993;3:1913-1919. 8338923 10.1681/ASN.V3121913

[21] Soupart A Silver S Schrooeder B Sterns R Decaux G. Rapid (24-hour) reaccumulation of brain organic osmolytes (particularly myo-inositol) in azotemic rats after correction of chronic hyponatremia. J Am Soc Nephrol. 2002;13:1433-1441. 12039971 10.1097/01.asn.0000017903.77985.cd

[22] Oo TN Smith CL Swan SK. Does uremia protect against the demyelination associated with correction of hyponatremia during hemodialysis? A case report and literature review. Semin Dial. 2003;16:68-71. 12535304 10.1046/j.1525-139x.2003.03015.x

[23] Dhrolia MF Akhtar SF Ahmed E Naqvi A Rizvi A. Azotemia protects the brain from osmotic demyelination on rapid correction of hyponatremia. Saudi J Kidney Dis Transpl. 2014;25:558-566. 24821152 10.4103/1319-2442.132183

[24] Miller GM Baker HL Jr Okazaki H Whisnant JP. Central pontine myelinolysis and its imitators: MR findings. Radiology. 1988;168:795-802. 3406409 10.1148/radiology.168.3.3406409

[25] Brunner JE Redmond JM Haggar AM Kruger DF Elias SB. Central pontine myelinolysis and pontine lesions after rapid correction of hyponatremia: a prospective magnetic resonance imaging study. Ann Neurol. 1990;27:61-66. 2301929 10.1002/ana.410270110

[26] Sterns RH Cappuccio JD Silver SM Cohen EP. Neurologic sequelae after treatment of severe hyponatremia: a multicenter perspective. J Am Soc Nephrol. 1994;4:1522-1530. 8025225 10.1681/ASN.V481522

[27] Sterns RH Riggs JE Schochet SS Jr. Osmotic demyelination syndrome following correction of hyponatremia. N Engl J Med. 1986;314:1535-1542. 3713747 10.1056/NEJM198606123142402

[28] Soupart A Ngassa M Decaux G. Therapeutic relowering of the serum sodium in a patient after excessive correction of hyponatremia. Clin Nephrol. 1999;51:383-386. 10404700

[29] Huang WY Weng WC Peng TI Ro LS Yang CW Chen KH. Central pontine and extrapontine myelinolysis after rapid correction of hyponatremia by hemodialysis in a uremic patient. Ren Fail. 2007;29:635-638. 17654329 10.1080/08860220701392314

[30] Peces R Ablanedo P Alvarez J. Central pontine and extrapontine myelinolysis following correction of severe hyponatremia. Nephron. 1988;49:160-163. 3380231 10.1159/000185044

[31] Tarhan NC Agildere AM Benli US Ozdemir FN Aytekin C Can U. Osmotic demyelination syndrome in end-stage renal disease after recent hemodialysis: MRI of the brain. AJR Am J Roentgenol. 2004;182:809-816. 14975990 10.2214/ajr.182.3.1820809

